# "The Hospital" Medical Book Supplement—No. XXXV

**Published:** 1910-10-15

**Authors:** 


### "THE HOSPITAL"
### Medical Book Supplement No. XXXV.

CONTENTS.

| | PAGE |
|---|---|
| **Medicine—** | |
| Colds and Influenza: their Prevention and Cure ... ... ... | 75 |
| A Practical Guide to the Newer Remedies ... ... ... ... | 75 |
| Diseases of the Skin; including Radio-therapy and Radium-therapy ... ... ... ... ... ... ... ... ... | 75 |
| **Pharmacy—** | |
| The Extra Pharmacopœia of Martindale and Westcott ... ... | 76 |
| **Anatomy—** | |
| Cunningham Manual of Practical Anatomy ... ... ... | 76 |
| **Hospitals—** | |
| The History of St. George's Hospital ... ... ... ... ... ... | 76 |
| **Miscellaneous—** | |
| Spiritism and Insanity ... ... ... ... ... ... ... | 77 |
| How to Become a Pharmacist ... ... ... ... ... ... | 77 |
| City and County of Bristol, 1909 ... ... ... ... ... | 77 |
| Philosophies ... ... ... ... ... ... ... ... | 78 |
| Popular Drugs: Their Use and Abuse ... ... ... ... | 78 |
| The Nursery Nurse's Companion ... ... ... ... ... | 78 |
| Practical Physiological Chemistry ... ... ... ... ... | 78 |
| St. Louis: Its History and Ideals ... ... ... ... ... | 78 |

### MEDICINE.

Colds and Influenza: Their Prevention and Cure. By J. Stenson Hooker, M.D., L.R.C.P., L.R.C.S., etc. (London: The Annals of Psychical Science, Ltd. 1910. Price 3d.)

One has but to keep one's eyes open when rubbing shoulders with the crowds that throng any big city of this England of ours to see that at least a fair percentage indulge too freely in the pleasures of the table. Increasing girth may make oneself conscious of the fact in one's own case, and yet it has probably never entered one's head that overfeeding, coupled with even a modicum of alcoholic refreshment, is the causal agent of cold and influenza! Still, such is the dictum of Dr. Hooker, who claims to have proved it in his own and other cases. Microbes are but secondary causes, the real cause being our own indulgence. It will not be long, he maintains, before the possession of a cold will seem a shameful thing to its victim, instead of making him, as now, an object of pity. Therefore avoid colds by eating less, and so prevent the tissues becoming clogged. Microbes cannot live unless they have the suitable culture-medium supplied by excess. No fancy diets are necessary, but alcohol should be avoided, and as much fresh air as possible taken into the lungs. Cold, draughts, and damp will then have no terrors, for a cold is here to-day and gone to-morrow when the individual is prepared. In most cases a trial of the preventive measures would not, we think, lead to starvation, for undoubtedly most of us eat too much. We have no personal experience of the remedy, so cannot speak of it at first hand. Still, if the prevention of colds is as simple a matter as the author makes out, by all means let the remedy be tried, even though the Meat Trust be ruined in the process.

A Practical Guide to the Newer Remedies. By J. M. Fortescue-Brickdale, M.A., M.D. Oxon. (Bristol: John Wright and Sons, Ltd. Price 5s. net.)

In view of the profusion of new remedies and proprietary drugs which are at present advertised so largely, each claiming to be superior to its rivals and better than the parent drug from which it is derived, it would scarce seem a matter of wonder if the busy practitioner were to vary slightly the phrase of poor Mercutio and mutter "A plague on *all* your houses," and henceforth confine his attention to drugs with the uses of which he is familiar. Such an attitude, however excusable, will not perhaps suit his patients, some of whom have leisure to read the literature of such new drugs, and may demand to know why they have not been treated with them. In such an event. Dr. Fortescue-Brickdale's guide will help the harassed practitioner. All the more important new drugs are dealt with in some detail and their value as remedies discussed with impartiality. Iodine, bromine, and sulphur compounds are first discussed, then those of arsenic, phosphorus, bismuth, and iron. A chapter on drugs acting on the intestines follows next, after which comes one on those acting on the circulation. Hypnotic, anæsthetic, and antipyretic drugs are then treated of in successive chapters, the volume being brought to a close with some remarks on certain specific remedies. The book is well arranged and contains much information that will be of interest to the profession at large, but perhaps most of all to those members of it who have had few opportunities of putting these newer remedies to a practical test, and who wish for information upon them before using them.

Diseases of the Skin: Including Radio-therapy and Radium-therapy. By Professor E. Gaucher. Translated and Edited by C. F. Marshall, M.D., F.R.C.S. (London: John Murray. 1910. Pp. 460. Price 15s. net.)

The French School of Dermatology has been so extremely prominent in the great progress which this science has made within the last twenty years, that an English translation of a standard French work on skin diseases is an event to be welcomed. The first thing that strikes the reader of this book is the vast difference in arrangement, style, and thought, between it and any representative English text-book—for instance the wholly admirable monograph by Sir Malcolm Morris. The French author displays more ingenuity, more imagination perhaps, and undoubtedly pursues classification further. But as a working guide to English medical practitioners his book cannot compare with that of their own countryman. It is, nevertheless, extremely interesting as showing points of view with which we in this country are unfamiliar; many of these are illuminating to a degree which well repays the time spent in perusing these pages.

One of the most noteworthy features of the book is the very large number of obscure and rare skin-diseases which are described, some of them not even mentioned in British text-books, as, for instance, carathès. The amount of space devoted to these dermatological curiosities is distinctly out of proportion to their importance from a practical standpoint. On the other hand, in dealing with the commoner rashes, there are occasionally to be found omissions which are quite astonishing. Under drug eruptions, for instance, no mention is made of ether rash and enema rash, probably the two commonest medicamentous skin eruptions that exist; nor is either entered in the index, so it is fair to conclude that both are entirely omitted. The sections on radio-therapy and radium-therapy are well abreast of the latest researches. It is interesting to read that the author has a very low estimate of the former for rodent ulcer, epithelioma, etc.; though he describes it in detail because, as he says, it is the fad of the moment. Of radium-therapy he has a much higher opinion, thus confirming the views of Wickham and Degrais of the Paris school. The sections on syphilis are among the best in the book, as might be expected from the interest which French clinicians have always displayed in research into this important subject. Electric treatment is highly extolled for lupus erythematosus, a lesion which is described as tuberculous by nature; high-frequency discharge with coincident scarification is regarded as the most satisfactory line of treatment. Carbonic-acid snow is not mentioned, either for this complaint or, as far as can be seen, for any other disease for which cauterisation is required. The illustrations are photographic almost throughout. Though many of these photographs are extremely good, they do not convey the appearances of eruptions in so satisfactory a way as can be done by drawings in colour or in black and white. The indexing of the volume has been done in an admirably thorough and complete manner.

### PHARMACY.

The Extra Pharmacopœia of Martindale and Westcott. Revised by W. Harrison Martindale, Ph.D., F.C.S., and W. Wynn Westcott, M.B.Lond., D.P.H. (London: H. K. Lewis. 1910. Pp. 1054. Price 12s. net.)

Owing to the space required for the description of new preparations put on the market during the past two years, it has been found necessary, on the appearance of a new edition, to change the appearance of this well-known work. It is now somewhat larger in dimensions than its predecessors, though, if anything, less bulky. New chapters have been devoted to lactic acid bacilli, organic arsenic compounds, iontophoresis, and radium, as well as to patent and proprietary medicines. A complete account of the Wassermann test is given, and the chapter on trypanosomiasis has been brought up to date. An account is also given of some work of one of the authors with reference to flavouring agents. He has attempted to simplify the preparation of these agents, while at the same time rendering them more useful to disguise the taste of nauseous mixtures. The volume is as useful as its predecessors, and contains a mine of information on all subjects connected directly and remotely with the subject of pharmacy. With one exception it has yielded information with regard to all the proprietary and patent medicines for which we have sought in its pages, and that one exception was antikamnia, which we cannot find in the index.

### ANATOMY.

Cunningham Manual of Practical Anatomy. Two volumes. Revised by Arthur Robinson. (Henry Frowde, and Hodder and Stoughton. 1910. Price 10s. 6d. per volume.)

Cunningham is sufficiently well known to all medical practitioners and students to need no word of commendation or introduction. This, the fourth revised edition, has largely been amended by the late Professor Cunningham himself, and these revisions have been considerably extended by Professor Robinson, who is responsible for the new edition as it appears. Among the additions are a glossary of the Basle nomenclature terms, a very useful innovation in a text-book such as this, for the Basle nomenclature is gradually being introduced into all the anatomical manuals, and although it is not yet compulsory in examinations its general use is only a matter of time. It is imperative for the student to master its intricacies as soon as possible, and it will be as well for the senior student and the practitioner, who have already learned their anatomy, to learn the new terminology as well. For that purpose we can advise no better book than Cunningham. It is essentially practical, and its beautifully drawn illustrations, which show actual dissections and not fanciful diagrams, are a real help to the reader. This edition, it is worth while pointing out, contains several new illustrations, while a few of the old ones have been re-drawn and coloured. The section on the male perineum, for instance, has been much improved by the addition of these revised blocks. That of the pelvis, in our opinion hitherto not the best part of the book, has been considerably bettered by the inclusion of further illustrations. The old division, which has proved itself so highly popular with students, has been maintained, and for regional anatomy we know of no dissecting-room manual that can compare with these two volumes. The second volume, dealing with the head and neck, shows less alteration, but it was always perhaps the better volume of the two, and still maintains its position as one of the most lucid expositions of the anatomy of the thorax and head and neck. The chapter on the brain and that on the dorsal aspect of the trunk are models of what such articles should be. We have no hesitation in recommending this book to everyone who wishes to possess a thoroughly sound, practical, and up-to-date manual of anatomy, of a handy size, well printed, and strongly bound, and, above all, cheap.

### HOSPITALS.

The History of St. George's Hospital. By G. C. Peachey. Part II. (London: J. Bale, Sons and Danielsson. 1910. Price 2s. 6d. net.)

In this, the second, part of Mr. Peachey's important history of his old hospital he enters with extreme thoroughness into the early history of the land which now constitutes Belgravia, Knightsbridge, Hyde Park, and the neighbourhood; and explains the exact ownership, uses, and thoroughfares at the time when Lanesborough House was converted into St. George's Hospital; of the tract which is now enclosed by Sloane Street, St. George's Place, Grosvenor Place, and Ebury Street. To anyone acquainted with the story of the evolution of our modern London it is most fascinating thus to trace out, as Sir Walter Besant loved to do, the origin of street names, of divisions of property, of old buildings, and the like. Mr. Peachey spends much space over this theme, and no one is likely to grudge it him. The antiquarian knowledge he displays is most interestingly set forth, and his description of London life in the early eighteenth century, if rather a side issue of his main theme, is quite excellent. Finally, leaving the general geography of Knightsbridge (which is, we learn, a corruption of its original name of Kingsbridge), he deals with the site of the hospital itself, and with the original building which stood on it. The story of the structure itself is told down to 1826, when the hospital was rebuilt. The work is maintaining the very favourable impression which the first part created, and the remaining parts will be awaited with interest.

### MISCELLANEOUS.

Spiritism and Insanity. By Dr. Marcel Viollet, Physician to the Lunatic Asylums, Paris. (London : Swan Sonnenschein and Co., Ltd. No. I. of the "Library of Experimental Psychology and Metapsychism." Pp. 134 + x. Price 2s. 6d. net.)

The author of this short volume is a believer in spiritism, but his belief has not blinded his judgment to the extent of causing him to deny the existence of fraud and chicanery among the practitioners of its doctrines. Neither is he able to ignore the fact that among followers of the cult are to be found many whose minds are disordered to varying extents. He acknowledges that among mediums many of both these classes are to be met with. For the insane and those predisposed to insanity there is a danger both to themselves and to others in attending spiritistic *séances*. The danger to themselves is hallucination and delirium; to others the evolution of that delirium or of impulses and reactions of all kinds which the insane owe to their mentality diseased from other causes. The author concludes that spiritism must be freed of fraud and lunatics before it will be allowed to take the place it deserves as a recognised science. The book deals with the various types of mental instability likely to be met with among the ranks of spiritists. These are classified and described at some length, histories of some of the more typical cases being given. The work of the anonymous translator has on the whole been well done, but there has been in places a tendency to assume that some French words are best translated by writing them down directly into English, thus "diagnostique" becomes "diagnostic" in a place where diagnosis obviously is meant, and "céphalée" becomes "cephalus" instead of headache. The book will serve as a useful introduction to the subject with which it deals.

How to Become a Pharmacist. A Guide to Apprenticeship in Pharmacy and the Examinations of the Pharmaceutical Society of Great Britain. Edited by John Humphrey. (London : The Pharmaceutical Press. 1910. Pamphlet of 45 pages. Price 1s. net.)

To obtain the qualification of "chemist and druggist" in Great Britain it is necessary to be twenty-one years of age, to have passed an approved preliminary examination, to be registered as an "apprentice or student," to have had three years' practical experience in the translation and dispensing of prescriptions, and to have passed the Minor Examination of the Pharmaceutical Society. On the fulfilment of these conditions the student is registered as a "chemist and druggist," and is eligible for election as a member of the Pharmaceutical Society. A second examination, known as the "Major," entitles the person who has passed it to registration as a "pharmaceutical chemist." The pamphlet before us aims at giving would-be pharmacists good advice as to how to set about obtaining the necessary qualification. The advantages of pharmacy as a career are set forth, some remarks are made on the law of apprenticeship, and many useful hints are thrown out to guide the novice in his career as a student. The full syllabuses of both Minor and Major examinations are given, the numerous scholarships and prizes open to students are described, and details of the study necessary to obtain them stated in full. Four Appendices bring the pamphlet to a close. The first deals with text-books for students, the second with pharmaceutical qualification in Ireland (which is not reckoned as part of Great Britain, and in which the qualifications of the English Pharmaceutical Society are not recognised), the third with degrees in pharmacy granted by the Universities of Manchester and Glasgow, and the last with poisons on the schedule. The pamphlet should prove extremely useful to all desirous of knowing how to become a dispensing chemist.

City and County of Bristol, 1909. Annual Report of the Medical Officer of Health and of the General Medical Superintendent of the City Hospitals. (Bristol : Bennett Brothers, Ltd., 1910.)

The city of Bristol stands on an area of 17,289 acres, and has a population, estimated at the middle of 1909, of 377,642 persons. The births registered in 1909 were 8,507, of which 296, or 3.4 per cent., were returned as illegitimate. The birth-rate was 22.5 per thousand, as compared with 23.0 for the previous year, and with 25.7, the average for 1909 in the seventy-six great towns. The excess of births over deaths amounted to 3,638. Within the borough 2,670 marriages took place during the year, the annual marriage rate being 7.0 per thousand, as compared with 7.5 the year previous. Deaths amounted to 4,869, of which sixty-nine were returned as deaths of illegitimate children. The general death-rate was 12.89 per thousand, as compared with 13.76 for 1908, and 14.7 for the great towns. The rate 12.89 is the lowest ever recorded for the city. The infantile death-rate worked out at 101.0 per thousand, compared with 125.8 for 1908, and 118 for the great towns. The zymotic death-rate was 0.9 per thousand, as compared with 1.4 for the great towns. Thirty-nine cases of small-pox were notified, of which nine died, giving a case-mortality of 23 per cent. The type of the disease was extremely virulent, three deaths occurring from hæmorrhagic small-pox, all in unvaccinated children, three other cases of those notified being also hæmorrhagic in type, but not fatal. Nearly everyone who came in contact with the initial case at Wick, in the County of Gloucester, contracted the disease, and this despite recent vaccination in some cases. Among the vaccinated, however, symptoms were remarkably mild. This outbreak cost the city £829 17s. 11d. The medical officer remarks on the opposition to vaccination exhibited both without and within the Health Committee. One unexpected result of the heated discussions of that body was that private firms undertook on their own responsibility the duty which the public vaccination authority refused to perform, with the result that a far larger amount of revaccination was secured in the city than upon any previous occasion within the Medical Officer's knowledge, and this was a valuable asset towards safety. Medical practitioners reported 3,914 primary and 18,903 revaccinations. These returns came from 186 practitioners, fifty-five making no returns, and nine keeping no records. Scarlet fever cases amounted to 692, with twelve deaths and a case-mortality of 1.7 per cent. Enteric fever caused twelve deaths out of sixty-six cases, a case-mortality of 18 per cent. Diphtheria gave a case-mortality of 7.7, there being fifty-five deaths out of 712 cases. Infantile diarrhœa was responsible for 116 deaths. Three deaths occurred out of 199 cases of erysipelas. Seventeen died out of thirty-six patients suffering from puerperal fever. Measles accounted for ninety deaths, whooping-cough for fifty-six. Influenza was credited with twenty-seven deaths, cerebro-spinal fever with four. The fatalities from phthisis and other tuberculous diseases amounted respectively to 391 and 133. Under the voluntary system 395 cases were notified, and 173 under the Public Health (Tuberculosis) Regulations 1908. Of seven samples of water sent by the medical officer for analysis, two showed sewage pollution, one showed surface drainage, one showed part pollution, one showed intermittent pollution, and one fell within the limits of sanitary purity. The reports of the chief inspector of nuisances, the inspector of bakehouses, and the factory inspector call for no remark.

Philosophies. By Ronald Ross. (John Murray. 1s. net.)

When a little book of verses calls attention so loudly on its title-page to the scientific degrees and other honours of its author, as "Philosophies," by Ronald Ross, F.R.C.S., D.Sc., LL.D., F.R.S., C.B., proceeds to do, we hardly expect to find much poetry inside it. The artist does not do this sort of thing; he has some sense of literary good manners, not to say some sense of humour, both of which are lacking here. Fancy "Endymion" appearing, as on these lines it might have done, with "by John Keats, M.L.S.S.A., L.S.A.," on the title-page; but then Keats was a poet, which whatever else Major Ross may be he is not and never will be. His work lies elsewhere, not with metre, but with malaria and microbes; not with prosody, but with preventive medicine. The personal note in the "poems" is struck continually: "Truth, whom I hold divine," thus the man of many titles patronises in verse what poets have ventured to praise only humbly. The best of these bad verses are rhetorical merely, and rhetoric has been described by one who knew as "the will doing the work of the imagination." Let Major Ross think over this, and then return to his own sphere of scientific research. It is in poetry not in India that he is really "in exile," and the prose of his preface is the happiest part of his book.

Popular Drugs: Their Use and Abuse. By Sydney Hillier, M.D. (London: T. Werner Laurie. 1910. Pp. 192. Price, 3s. 6d. net.)

This monogragh, dealing concisely with the evils of indiscriminate drugging, can be heartily recommended to the layman, for whom it was obviously penned. Alcohol, tea, coffee, cocoa, and tobacco are dealt with in addition to those substances which the public is more apt to look upon as drugs than these; in fact, the term "drug" is used in its widest sense. While telling us practically nothing new, the author puts his case clearly and in an interesting manner. The short historical notes on each drug wherewith he prefaces his remarks on its effects add much to the "readableness" of his work. He attacks his subject from the point of view of the average physician. His advice is sensible, and he has no axe to grind on behalf of teetotalism, for he does not attribute to drink all the ills that flesh is heir to. His remarks on the so-called "safe" hypnotics such as veronal, trional, tetronal, etc., are to the point, and the public would do well to take heed of his warning as to the dangers of using diachylon pills and other lead preparations often taken as abortificients. It is unfortunate that the very class most in need of this warning is the one least likely to come across this book. The volume is marred by a number of misprints, *e.g.* on pp. 31, 58, and 67.

The Nursery Nurse's Companion. By Honnor Morten. (London: Mills and Boon. 1910. Pp. 140. Price 1s. net.)

The first few chapters of the book are devoted to methods of training of the nurse. Mention is made of the various establishments, sectarian and non-sectarian, where systematic education can be obtained in the art of looking after children. The remainder of the volume deals with the care of the child, its diet at various ages, its clothing, its management, games, ailments and accidents. We are glad to notice that under the somewhat euphemistic heading of "bad habits," the nurse's attention is called to practices which, if allowed to continue unchecked, will be a source of trouble, if not of actual misery in later life. An observant and tactful woman can do much to repress such habits without calling the child's attention to their sexuality or making it self-conscious. These practices are started in innocence, and continued in innocence, until a habit is acquired which necessitates a great deal of will power to overcome. The volume should be of value to the children's nurse.

Practical Physiological Chemistry. By R. H. Aders Plimmer, D.Sc. (London: Longmans, Green and Co. 1910. Pp. 270. Price 6s. net.)

Whether organic chemists will probe the secrets of physiology deeply enough to solve the mystery of life the future will decide; but few things bring home better the incessant advances which go forward in the preliminary medical sciences than to look through the pages of an up-to-date text-book, elementary in design though it may be. Dr. Plimmer's book, for instance, begins with the familiar and elementary lessons in practical physiological chemistry which one learnt not so very many years ago in student days. But soon he begins to deal with reactions and processes, then undreamt of, which are now the commonplaces of the demonstrator and the average pupil. Even the list of reagents necessary for the chemico-physiological laboratory of to-day occupies six large pages, and the precision with which estimations of every product of human metabolism can now be undertaken is astonishing. The most striking features of Dr. Plimmer's book are the lucidity of exposition, and the excellent arrangement. The student who works through the course here outlined must needs come to his clinical studies with very clear conceptions of those metabolic processes whose derangements he has next to study; and if in after-life the activities of a busy practice prevent him from practising physiological chemistry on any important scale, he will at least have an understanding of the subject which cannot fail to benefit his patients.

St. Louis: Its History and Ideals. By Philip Skrainka, M.D. (St. Louis: The American Medical Association. 1910. Pp. 172.)

This interesting little volume gives the history of St. Louis from its foundation to the present day. Originally a trading-post formed by Pierre Laclede Liguest in 1763, it prospered greatly, and quickly became the centre of the peltry trade. With success came increase in population. Within a few years, however, the French possessions in America came under Spanish rule, an unpopular move which necessitated the presence of forty soldiers under Louis St. Ange de Bellerive in St. Louis. This officer was subsequently elected Governor of the town, and under him peace was maintained. His influence with Pontiac, chief of the Ottawas, secured peace between that tribe and the English. Under Spanish rule the liquor laws were stringent and effective. No liquor could be sold to the natives. After once again coming under French protection, Louisiana was finally ceded to the United States in 1803, from which time its development has been rapid. To-day it has a population of 700,000, whereas at the last census taken by the Spaniards in 1799 it had a population of 1,008, including fifty-three mulattoes, six free negroes, and 268 slaves. The little volume contains an account of the growth of the city, as well as of the history of its university and medical school, and is profusely illustrated with excellent photographs of public monuments, buildings, parks, and pleasure resorts both in the city and in its vicinity.

